# Blood correction reduces variability and gender differences in native myocardial T1 values at 1.5 T cardiovascular magnetic resonance – a derivation/validation approach

**DOI:** 10.1186/s12968-017-0353-7

**Published:** 2017-04-05

**Authors:** Jannike Nickander, Magnus Lundin, Goran Abdula, Peder Sörensson, Stefania Rosmini, James C. Moon, Peter Kellman, Andreas Sigfridsson, Martin Ugander

**Affiliations:** 1 Department of Clinical Physiology, Karolinska Institutet and Karolinska University Hospital, Stockholm, Sweden; 2 Department of Medicine, Unit of Cardiology, Karolinska Institutet and Karolinska University Hospital, Stockholm, Sweden; 3 Institute of Cardiovascular Science, University College London, London, UK; 4 National Heart, Lung, and Blood Institute, National Institutes of Health, Bethesda, MD USA

**Keywords:** Native T1, T1 Mapping, Post-processing, Quantification, Precision

## Abstract

**Background:**

Myocardial native T1 measurements are likely influenced by intramyocardial blood. Since blood T1 is both variable and longer compared to myocardial T1, this will degrade the precision of myocardial T1 measurements. Precision could be improved by correction, but the amount of correction and the optimal blood T1 variables to correct with are unknown. We hypothesized that an appropriate correction would reduce the standard deviation (SD) of native myocardial T1.

**Methods:**

Consecutive patients (*n* = 400) referred for CMR with known or suspected heart disease were split into a derivation cohort for model construction (*n* = 200, age 51 ± 18 years, 50% male) and a validation cohort for assessing model performance (*n* = 200, age 48 ± 17 years, 50% male). Exclusion criteria included focal septal abnormalities. A Modified Look-Locker inversion recovery sequence (MOLLI, 1.5 T Siemens Aera) was used to acquire T1 and T1* maps. T1 and T1* maps were used to measure native myocardial T1, and blood T1 and T1*. A multivariate linear regression correction model was implemented using blood measurement of R1 (1/T1), R1* (1/T1*) or hematocrit. The correction model from the derivation cohort was applied to the validation cohort, and assessed for reduction in variability with the F-test.

**Results:**

Blood [LV + RV] mean R1, mean R1* and hematocrit correlated with myocardial T1 (Pearson’s r, range 0.37 to 0.45, *p* < 0.05 for all) in both the derivation and validation cohorts respectively, suggesting that myocardial T1 measurements are influenced by intramyocardial blood. Mean myocardial native T1 did not differ between the derivation and validation cohorts (1030 ± 42.6 ms and 1023 ± 45.2 ms respectively, *p* = 0.07). In the derivation cohort, correction using blood mean R1 and mean R1* yielded a decrease in myocardial T1 SD (45.2 ms to 36.6 ms, *p* = 0.03).

When the model from the derivation cohort was applied to the validation cohort, the SD reduction was maintained (39.3 ms, *p* = 0.049). This 13% reduction in measurement variability leads to a 23% reduction in sample size to detect a 50 ms difference in native myocardial T1.

**Conclusions:**

Correcting native myocardial T1 for R1 and R1* of blood improves the precision of myocardial T1 measurement by ~13%, and could consequently improve disease detection and reduce sample size needs for clinical research.

## Background

Parametric pixel-wise mapping has been developed to quantitatively measure and image the longitudinal magnetic relaxation time (T1) [1]. Native myocardial T1 mapping has shown diagnostic promise for differentiating healthy myocardium from various pathologies including acute myocardial infarction [2], myocarditis [3, 4], amyloidosis [5], edema [6], Anderson-Fabry disease [7] and other non-ischemic diseases [8, 9]. Reference values for native myocardial T1 in ischemic and several non-ischemic heart diseases have been reported, with cutoff values for age, and sex [10], which suggests that native myocardial T1 mapping is a robust diagnostic tool for myocardial tissue characterization in clinical use [5, 11, 12].

However, the normal myocardial blood volume (MBV) averages approximately 6% of the myocardium [13, 14]. The T1 value of blood is higher than the T1 value of the myocardium, thus the T1 value of the myocardium is expected to be influenced by both the T1 value, and the amount of blood in the myocardium. The partial volume effect describes the concept that a single voxel may contain tissue of different characteristics, whereas the pixel value for that voxel will represent a combination of these contents [15]. Furthermore, the myocardial T1 values reflect a volume of tissue consisting of myocytes, extracellular connective tissue, nerves, blood vessels and the blood therein [16, 17]. Therefore, myocardial T1 values should relate to the summed composition of the tissue, including the microcirculation. The T1 values of blood have been shown to vary due to a number of factors like hematocrit [18], sex [10], age [19], and oxygen pressure [18, 20] and variations in these factors may influence the interpretation of myocardial T1 values, and obscure the true T1 value of the myocardium.

Approaches for correcting for myocardial blood volume have been reported [21, 22]. Reiter et al. showed that myocardial T1 differences by age and sex were eliminated when corrected for T1 measured in the blood pool of the left ventricle (LV) [22]. This correction could therefore decrease the variability of native myocardial T1 between patients, thus improving the clinical accuracy. Therefore, the purpose of this study was to identify a blood correction model, with cardiovascular magnetic resonance (CMR) available measurements, which in a future implementation could allow automated inline blood-corrected T1 maps, and validate this model in a separate clinical cohort. A technical aspect of an automated method for blood correction is blood segmentation. Therefore, the mean blood T1 values of both ventricles (LV and right ventricle (RV)) were acquired. Blood T1* measurements were also investigated. Since there is no reference method for measuring myocardial T1 values, we hypothesized that blood correction could reduce the variability in native myocardial T1 values in a clinical population, measured as a decrease in standard deviation (SD).

## Methods

### Study population

**Fig. 1:**
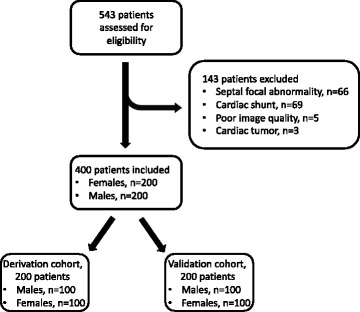
Patient selection

Consecutive patients referred for diagnostic CMR for known or suspected heart disease at Karolinska University Hospital, Stockholm, Sweden, were retrospectively enrolled between 2014-01-02 and 2015-08-07. Inclusion was terminated when a total of 200 males and 200 females were included, respectively. A total of 543 patients were assessed for eligibility. Exclusion criteria included focal septal myocardial pathologies, cardiac shunts, poor image quality or presence of cardiac tumors, see Fig. 1. Included patients were randomly assigned to either the derivation or the validation cohort. Hematocrit was determined by venous blood sampling prior to CMR, as a part of the clinical routine. Venous blood was drawn with a syringe from an intravenous access cannula, and analyzed by an iSTAT Handheld Blood Analyzer®, using a CHEM8+ cartridge (Abbot Point of Care, Princeton, USA). The study was approved by the local research ethics committee, and all patients provided written informed consent.

Healthy volunteers (*n* = 77, mean age 49 ± 14 years, 49% males) were prospectively recruited between April 2014 and July 2015 through local advertising. None of the volunteers had any cardiovascular disease or diabetes. Five were on statins and one was on aspirin for primary prevention. All volunteers had a normal 12-lead ECG and all scans were reported as normal by experienced Level 3 CMR cardiologists. Imaging was performed on a 1.5 T MR scanner (Magnetom Avanto, Siemens, Erlangen, Germany) and a 32-channel cardiac phased array receiver coil was used. The imaging protocol included cines, native T1 mapping, T2 mapping, Late Gadolinium Enhancement (LGE) and post-contrast T1 mapping. For this study, only the native T1 and T1* maps were analysed. Whole blood for venous hematocrit was drawn in all subjects by venipuncture and analyzed as routine clinical samples using a Sysmex XE-2100 hematology analyzer (Sysmex, Kobe, Japan) [23].

### Image acquisition

All patient CMR scans were conducted on a 1.5 T scanner (Siemens Aera, Erlangen, Germany) with a phased-array 18-channel body matrix coil together with a spine matrix coil. All patients were examined in the supine position. CMR scans assessing LV function used retrospectively electrocardiographically gated balanced steady-state free precession (bSSFP) cine imaging covering the entire LV. Typical imaging parameters included flip angle = 61°, voxel size = 2.0x2.0x8.0 mm^3^, TR/TE = 37.05/1.19 ms, matrix size = 143x256 and field of view (FoV) 303x360 mm^2^.

T1 maps were acquired using an electrocardiographically gated Modified Look-Locker inversion recovery (MOLLI) sequence [24] with a 5(3)3 sampling scheme (Siemens WIP 1041). T1 maps were reconstructed using inline motion correction [25], and reconstruction output included a T1 error map, a T1* map and a T1 map. Typical image acquisition parameters were: steady-state free precession single-shot read out with a trigger delay to coincide with end-diastole, flip angle = 35°, matrix size = 144x256, TE = 2.6 ms, TR = 278 ms, FoV = 270x360 mm^2^, parallel acquisition technique (PAT) factor 2, number of inversion 2, data window duration 163 ms. Operators were allowed to perform standard cardiac planning adjusting for patient size, rendering a range of phase encoding lines 136–158, and FoV 241–384 x 300–410 mm^2^.

The healthy volunteers were imaged using a 5s(3s)3s MOLLI protocol. The acquisition parameters were: pixel bandwidth 977 Hz/pixel; echo time = 1.1 ms; flip angle = 35°; matrix size = 256x144; slice thickness = 6 mm. Motion correction and a non-linear least-square curve fitting were performed with the set of images acquired at different inversion times to generate a pixel-wise T1 map and T1* map.

### Image analysis

**Fig. 2:**
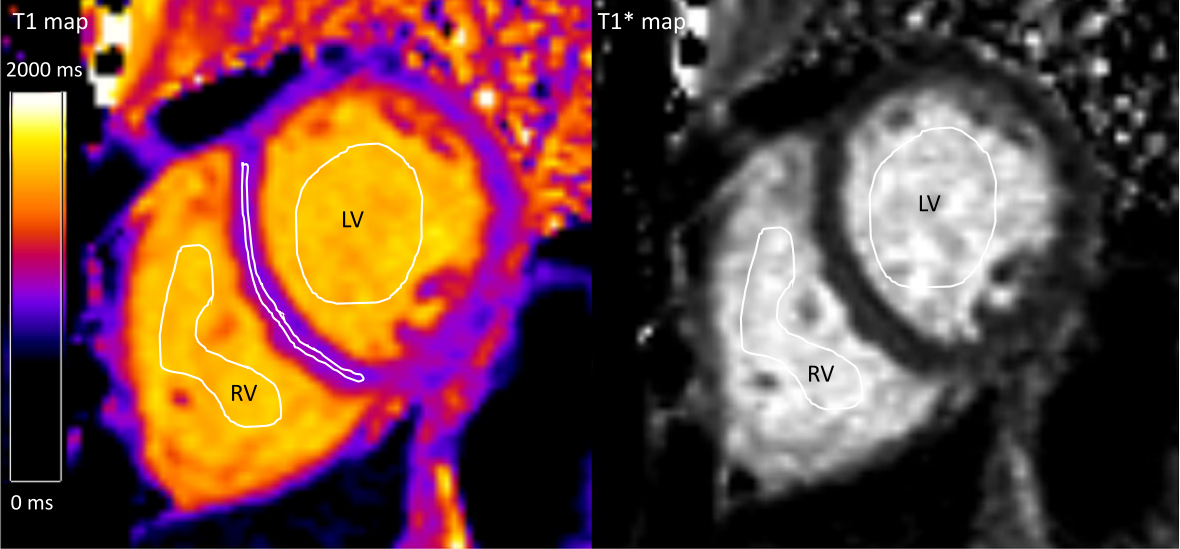
Short-axis T1 and T1* maps. The figure shows regions of interest (ROI) drawn for native myocardial T1 values of the mid-mural septum, LV blood pool T1 and T1* values, and RV blood pool T1 and T1* values

LV volumes, ejection fraction (EF) and myocardial mass were quantified using the SyngoVia Software, VA30 (Siemens, Erlangen, Germany). Body surface area (BSA) was calculated with the Du Bois formula [26]. Volumetric measurements and myocardial mass were indexed to BSA. Native myocardial T1 values were measured by one observer by manually delineating a region of interest (ROI), conservatively placed, to avoid partial volume effects, in the mid-mural third of the septum of a mid-ventricular short-axis T1 map using a clinical work station (IDS7, Sectra, Linköping, Sweden). Blood T1 and T1* values were measured in the blood pool of both ventricles as the mean value of two manually delineated ROIs in the corresponding mid-ventricular short-axis T1 and T1* maps. The ROIs were delineated as large as possible without including papillary muscle and trabeculae, see Fig. 2. Myocardial wall thickness was measured in the septum in the corresponding bSSFP cine short-axis slice, in end-diastole. Mean blood T1 and T1* values were calculated as the average of blood T1 and T1* from the RV and the LV, and converted to the T1 relaxation rate (R1 = 1/T1). This yielded mean blood R1 and mean blood R1*. R1, as opposed to T1, is linearly related to the concentration of differing T1 species such as contrast agent or blood in the setting of the current study.

A linear correlation between septal myocardial T1 and blood measurements was assumed. T1 was corrected using the equation, Equation 1:

$$ \mathrm{T}{1}_{\mathrm{corrected}}=\mathrm{T}{1}_{\mathrm{uncorrected}}+\mathrm{constant}\cdot \left({X}_{\mathrm{mean}}-{X}_{\mathrm{patient}}\right) $$

where *X* is the blood measurement of mean R1, mean R1* or hematocrit, mean is the mean for the patient cohort, and the constant was calculated as the slopes of linear regression between myocardial T1 and the blood measurements.

The T1 maps from the healthy volunteers were analysed using CVI42 software (Circle Cardiovascular Imaging Inc.,Version 5.1.2 [303], Calgary, Canada) by one observer. Global myocardial T1 values were acquired by carefully delineating the endo- and epicardial borders and eroding 10% to acquire the middle 80% of the myocardium. ROIs were drawn in the LV and RV blood pools of the mid-ventricular short-axis slice of the native T1 and T1* maps, and care was taken to avoid papillary muscles.

### Statistical analysis

Continuous variables were reported as mean and SD. Statistical analysis was performed using Microsoft Excel (Microsoft, Redmond, Washington, USA), and statistical testing was performed using Statistical Product and Service Solutions® (IBM SPSS Statistics 23, IBM, New York, USA). Statistical significance was defined as *p* < 0.05.

Multivariate linear regression was performed for slopes used for correction of native myocardial T1 for combinations of blood characteristics in the derivation cohort. The blood correction model from the derivation cohort was applied to the validation cohort, and the healthy volunteers respectively. The relationships between corrected and uncorrected native myocardial T1 values were investigated by the SD of the mean native myocardial T1, and evaluated for differences with the F-test. Mean values were compared by using the paired or unpaired *t*-test as appropriate in normally distributed data, and Mann–Whitney *U*-test in data with non-normal distribution. Sample size calculations were performed using Microsoft Excel.

## Results

### Study population

Baseline characteristics of the derivation and validation cohorts are shown in Table 1. Mean uncorrected native myocardial T1 was 1030 ± 42.6 ms in the derivation cohort, 1023 ± 45.2 ms, (*p* = 0.07) in the validation cohort, and 1027 ± 37.5 ms in the healthy volunteers. Hematocrit range for all 400 patients was 25 to 57%. There was no difference in characteristics between the derivation and validation cohorts, except for LV stroke volume (SV) and indexed LVSV (LVSVI), which was lower in the derivation cohort, albeit with a small magnitude of difference. There was no difference in characteristics between the females in the validation and derivation cohorts shown in Table 2. There was no difference between the males in the derivation and validation cohorts, except for LVSV and LVSVI, Table 3.

**Table 1:** Baseline characteristics of the derivation and validation cohorts

Characteristic	Derivation cohort (*n* = 200)^a^	Validation cohort (*n* = 200)^a^	*p*-value
Age (years)	51 ± 18	48 ± 17	0.14
Female sex, n (%)	100 (50%)	100 (50%)	1.0
Height (cm)	173 ± 14	174 ± 10	0.44
Weight (kg)	77 ± 16	78 ± 18	0.79
BSA (m^2^)	1.9 ± 0.3	1.9 ± 0.2	0.95
LVEDV (ml)	180 ± 63	183 ± 63	0.56
LVEDVI (ml/m^2^)	94 ± 29	95 ± 32	0.61
LVESV (ml)	91 ± 57	88 ± 57	0.81
LVESVI (ml/m^2^)	47 ± 28	46 ± 29	0.95
LVSV (ml)	89 ± 27	95 ± 25	0.02*
LVSVI (ml/m^2^)	47 ± 14	50 ± 11	0.01*
LVEF (%)	52 ± 12	54 ± 11	0.06
LVM (g)	138 ± 46	137 ± 53	0.75
LVMI (g/m^2^)	71 ± 19	72 ± 23	0.95
ECV (%)	28 ± 4	28 ± 4	0.51
Hematocrit (%)	40 ± 4	40 ± 5	0.71
Septal wall thickness (mm)	10 ± 2	10 ± 2	0.45
Myocardial T1 (ms)	1030 ± 43	1023 ± 45	0.07
Mean blood R1 (ms^−1^)	0.00064 ± 0.00004	0.00064 ± 0.00004	0.49
Mean blood R1* (ms^−1^)	0.00061 ± 0.00005	0.00061 ± 0.00005	0.98

Characteristics are given as means ± standard deviations, or percentages as appropriate. *p*-values refer to comparison of mean values between derivation and validation cohort. *marks significant result. ^a^Data missing for LV volumes, EF and mass (*n* = 2), and ECV (*n* = 4) in the derivation cohort. Data missing for ECV (*n* = 2) in the validation cohort

**Table 2:** Baseline characteristics of the females in the derivation and validation cohorts

Characteristic, females (*n* = 200)	Derivation cohort (*n* = 100)^a^	Validation cohort (*n* = 100)^a^	*p*-value
Age (years)	52 ± 18	49 ± 17	0.21
Female sex, n (%)	100 (100%)	100 (100%)	1.0
Height (cm)	165 ± 8	167 ± 7	0.17
Weight (kg)	69 ± 14	70 ± 16	0.89
BSA (m^2^)	1.8 ± 0.2	1.8 ± 0.2	0.77
LVEDV (ml)	157 ± 52	159 ± 49	0.48
LVEDVI (ml/m^2^)	89 ± 28	89 ± 26	0.47
LVESV (ml)	91 ± 57	88 ± 57	0.81
LVESVI (ml/m^2^)	47 ± 28	46 ± 29	0.95
LVSV (ml)	78 ± 21	83 ± 20	0.65
LVSVI (ml/m^2^)	45 ± 11	47 ± 11	0.16
LVEF (%)	53 ± 12	54 ± 11	0.44
LVM (g)	110 ± 26	112 ± 42	0.41
LVMI (g/m^2^)	62 ± 14	64 ± 22	0.49
ECV (%)	29 ± 4	29 ± 3	0.72
Hematocrit (%)	38 ± 4	38 ± 5	0.93
Septal wall thickness (mm)	9 ± 2	9 ± 3	0.45
Myocardial T1 (ms)	1046 ± 41	1040 ± 37	0.29
Mean blood R1 (ms^−1^)	0.00062 ± 0.00003	0.00062 ± 0.00003	0.58
Mean blood R1* (ms^−1^)	0.00059 ± 0.00004	0.00054 ± 0.00005	0.95

Characteristics are given as means ± standard deviations, or percentages as appropriate. *p*-values refer to comparison of mean values of derivation and validation cohort. ^a^Data missing for LV volumes, EF and mass (*n* = 2), and ECV (*n* = 4) in the derivation cohort. Data missing for ECV (*n* = 2) in the validation cohort

**Table 3:** Baseline characteristics of the males in the derivation and validation cohorts

Characteristic, males (*n* = 200)	Derivation cohort (*n* = 100)	Validation cohort (*n* = 100)	*p*-value
Age (years)	49 ± 18	47 ± 18	0.40
Female sex, n (%)	0 (0%)	0 (0%)	1.0
Height (cm)	181 ± 14	180 ± 8	0.81
Weight (kg)	86 ± 14	85 ± 16	0.84
BSA (m^2^)	2.0 ± 0.3	2.1 ± 0.2	0.77
LVEDV (ml)	204 ± 64	207 ± 68	0.67
LVEDVI (ml/m^2^)	99 ± 29	100 ± 36	0.96
LVESV (ml)	103 ± 62	101 ± 64	0.71
LVESVI (ml/m^2^)	50 ± 29	49 ± 34	0.56
LVSV (ml)	99 ± 27	106 ± 24	0.047*
LVSVI (ml/m^2^)	49 ± 16	52 ± 11	0.02*
LVEF (%)	51 ± 12	54 ± 11	0.06
LVM (g)	166 ± 45	162 ± 51	1.0
LVMI (g/m^2^)	80 ± 20	80 ± 21	0.78
ECV (%)	27 ± 3	27 ± 4	0.60
Hematocrit (%)	42 ± 4	43 ± 4	0.41
Septal wall thickness (mm)	11 ± 2	11 ± 2	0.58
Myocardial T1 (ms)	1015 ± 39	1005 ± 46	0.10
Mean blood R1 (ms^−1^)	0.00066 ± 0.00004	0.00066 ± 0.00003	0.57
Mean blood R1* (ms^−1^)	0.00063 ± 0.00004	0.00063 ± 0.00004	0.98

Characteristics are given as mean ± standard deviations, or percentages as appropriate. *p*-values refer to comparison of derivation and validation cohort. *marks significant results

### Myocardial T1 correlation

All the blood measurements correlated with uncorrected native myocardial T1 in both the derivation and validation cohorts, see Table 4. Myocardial wall thickness was not correlated with native myocardial T1, and was therefore not included in the multivariate analysis. Hematocrit had the weakest correlation in both the derivation and validation cohort, respectively. Mean R1 and mean R1* had the same correlation in the derivation cohort, whereas mean R1 alone had the best correlation in the validation cohort.

**Table 4:** Correlations and multivariate linear regression coefficients

Characteristic	Univariate r	Multivariate beta	Multivariate beta, final model	Global R^2^
Derivation cohort (n = 200)				0.26 (*p* < 0.001)
Hematocrit (%)	−0.37 (*p* < 0.001)	0.00 (*p* = 0.98)		
Mean blood R1 (ms^-1^)	−0.45 (*p* < 0.001)	−0.28 (*p* = 0.003)	−0.28 (*p* = 0.001)	
Mean blood R1* (ms^-1^)	−0.45 (*p* < 0.001)	−0.28 (*p* = 0.002)	−0.28 (*p* = 0.001)	
Septal wall thickness (mm)	0.07 (*p* = 0.33)	-	-	
Validation cohort (*n* = 200)				
Hematocrit (%)	−0.39 (*p* < 0.001)			
Mean blood R1 (ms^-1^)	−0.49 (*p* < 0.001)			
Mean blood R1* (ms^-1^)	−0.44 (*p* < 0.001)			
Septal wall thickness (mm)	0.07 (*p* = 0.32)	-	-	

All blood measurements correlated with myocardial T1, however hematocrit was not significant in multivariate regression analysis, and therefore not included in the final model.

### Myocardial T1 blood correction

**Fig. 3:**
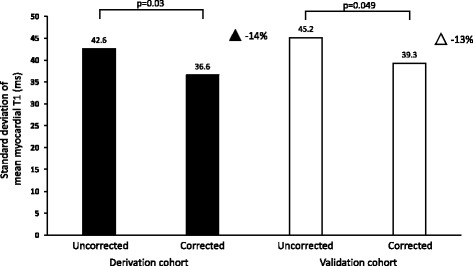
Standard deviation of corrected and uncorrected myocardial T1. The figure shows the SD of mean myocardial T1 in ms for uncorrected and blood corrected measurements, in the derivation cohort (black) and the validation cohort (white). The triangles denote reduction in variability in percent. *P*-values denote F-test

**Fig. 4:**
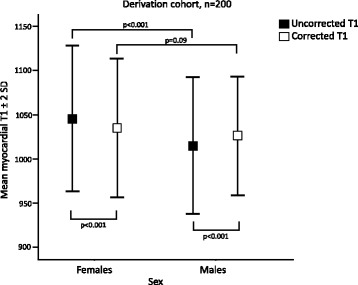
Mean myocardial T1 values for females compared to males, derivation cohort. The figure displays the mean ± 95% limits of agreement for females and males, prior to correction (black) and after blood correction (white). The mean myocardial T1 values differed between the sexes prior to blood correction, but the differences were eliminated after correction. *P*-values denote paired and unpaired *t*-test as appropriate

**Fig. 5:**
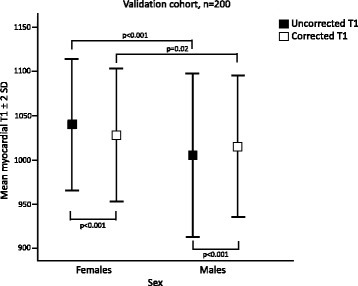
Mean myocardial T1 values for females compared to males, validation cohort. The figure displays the mean ± 95% limits of agreement for females and males, prior to correction (black) and after blood correction (white). The mean myocardial T1 values differed significantly prior to blood correction, and the differences were reduced, however not eliminated following correction. *P*-values denote paired and unpaired *t*-test as appropriate

Multivariate linear regression in the derivation cohort showed that the best correction model was a combination of mean R1 and mean R1* (R^2^ 0.26, *p* < 0.001), see Table 4. The constants from the regression model were used to correct native myocardial T1 values according to Equation 1. The blood correction model for mean R1 and mean R1* decreased the SD of myocardial T1 to 36.6 ms (*p* = 0.03) in the derivation cohort, see Fig. 3. The model also decreased the SD of myocardial T1 values in the validation cohort to 39.3 ms (*p* = 0.049), see Fig. 3. Mean uncorrected myocardial T1 in the derivation cohort was 1015 ± 38.6 ms for males and 1046 ± 41.1 ms for females (*p* < 0.001). The difference in mean myocardial T1 between males and females was eliminated by blood correction in the derivation cohort (1025 ± 33.3 ms vs 1035 ± 39.2 ms, *p* = 0.09), see Fig. 4. In the validation cohort, the mean uncorrected myocardial T1 was 1005 ± 38.6 ms for males and 1040 ± 37.0 ms for females (*p* < 0.001). The difference in mean myocardial T1 between males and females was reduced, but remained significant following blood correction, albeit by a small magnitude (1015 ± 40 ms vs 1028 ± 37.6 ms, *p* = 0.02), see Fig. 5. The range in myocardial T1 values in the derivation cohort decreased with 25% following blood correction (908–1183 ms vs 941–1146 ms).

### Myocardial blood correction in healthy volunteers

The SD of uncorrected myocardial T1 measurements of the healthy volunteers was 37.5 ms. The blood correction model for mean R1 and mean R1* did not decrease the SD (36.7 ms, *p* = 0.85). The mean uncorrected myocardial T1 in the healthy volunteers was 1012 ± 30.4 ms for males and 1042 ± 38.0 ms for females (*p* < 0.001). The difference in mean myocardial T1 between males and females was eliminated by blood correction in the healthy volunteers (1012 ± 33.7 ms vs 1023 ± 39.0 ms, *p* = 0.20).

### Differences in native myocardial T1

To ascertain if the partial-volume effect or hematocrit range contributed to the differences in native myocardial T1, the patients with the highest and lowest quartiles of wall thickness and hematocrit were examined in the derivation and validation cohorts, respectively. Patients with the thickest (>11.4 mm) and thinnest (<8.4 mm) myocardium had no difference in native myocardial T1 in the derivation cohort (1036 ± 44 ms vs 1028 ± 39 ms, *p* = 0.30). In the validation cohort, there was no difference in native myocardial T1 between patients with the thickest (>11.1 mm) and thinnest (<8.5 mm) myocardium (1026 ± 46 ms vs 1023 ± 38 ms, *p* = 0.76).

Patients with the highest (>43%) and lowest (<37%) hematocrit, differed significantly in myocardial T1 in the derivation cohort (1017 ± 44 ms vs 1054 ± 43 ms, *p* < 0.001). There was also a difference in myocardial T1 in the validation cohort, between patients with the highest (>44%) and lowest (<37%) hematocrit (993 ± 41 ms vs 1046 ± 30 ms, *p* < 0.001). These differences disappeared following blood correction, in both the derivation cohort (1033 ± 36 ms vs 1034 ± 36 ms, *p* = 0.87) and in the validation cohort (1012 ± 43 ms vs 1020 ± 31 ms, *p* = 0.35).

The reduction in SD allows for detection of differences with a smaller population in research studies. A reduction of 13% in SD reduces the sample size for identifying a 50 ms difference in myocardial T1 from 26 patients to 20 patients (23% reduction in sample size).

## Discussion

We have demonstrated that correcting for blood characteristics decreases the variability of native myocardial T1 in a clinical population, and reduces gender differences in healthy volunteers. Blood correction was achieved by using the average of LV and RV blood (mean blood R1 and mean blood R1*), resulting in a 13% reduction in variability for native myocardial T1 measurements in the validation cohort. Correction for hematocrit did not provide an incremental reduction in SD beyond image-based blood characteristics. The use of the LV and RV blood highlights the feasibility of implementing an automatic blood pool mask [27], in order for inline reconstruction of a blood corrected T1 map.

### Importance of blood correction

There are several physiological factors that influence the variation of blood T1 values, such as hematocrit [18], sex [10], age [19] and oxygen pressure [18, 20]. Also, technical aspects such as CMR magnetic field strength [12, 28, 29], off-resonance effects [30], and adiabatic inversion pulse imperfections [31] affect T1 values. Currently there is no reference standard for measuring myocardial T1 in vivo [32], and the extent of the effect of blood T1 on myocardial T1 is unknown. Furthermore, myocardial T1 values have been found to differ over the cardiac cycle, and this can be attributed to myocardial contraction compressing the vasculature [33] with reduced native myocardial T1 values during systole due to less blood (with longer T1) present in the myocardium [12]. Systolic readout has been proposed to reduce myocardial T1 variability by reducing partial volume effects since the myocardium is thicker in systole, and could theoretically include less intramyocardial blood [22, 34, 35]. Notably, in our data myocardial T1 values did not vary with end-diastolic myocardial thickness, but did vary with hematocrit. This suggests that measuring the native myocardial T1 values in a conservatively placed ROI in the midmural third of the septum in a clinical population reduces contamination from the blood pool. However, the results are surprising, considering that hematocrit did not independently contribute to native myocardial T1 values in the multivariate regression analysis. Hematocrit is not the only factor contributing to blood T1, and therefore the T1 of the blood is a better correction measure to use for native myocardial T1. Previous studies have assumed linear relationships between myocardial T1 values and blood T1 [22, 36]. In this study, we chose to correct for blood R1 and R1*. R1 is linearly related to the concentration of differing T1 species such as a contrast agent or blood, as opposed to T1. However, there could potentially be other relationships between native myocardial T1 values and other correction factors. This highlights the complexity of T1 mapping, but also stresses the importance of post-processing methods to increase precision and enhance clinical diagnostic use. To make the blood correction method clinically applicable we chose to correct for hematocrit, as this is determined prior to imaging in our clinical routine, for the purpose of performing extracellular volume fraction (ECV) mapping. Other parameters that could have been considered are ferritin or hepcidin. However, this was not performed in the current study. Furthermore, the blood in the RV is less oxygenated than in the LV, and therefore more paramagnetic than in the LV [18]. As the LV myocardium is directly perfused by the coronary arteries from the aorta, the use of mean blood T1 and T1* could potentially degrade the blood correction model. However, mean T1 and T1* values were chosen in order to make blood correction feasible by implementing an automatic (LV and RV) blood pool mask [27] for inline reconstruction of a blood corrected T1 map.

Furthermore, Reiter et al. [22] showed that sex differences in normal T1 values can be eliminated by blood correction. We also showed that sex differences were eliminated by blood correction in the derivation cohort, and in healthy volunteers. However, the sex differences in the validation cohort were reduced, but not eliminated following blood correction. This could be due to different distributions of physiological factors in different populations such as age, hematocrit, and oxygen pressure, as the means of these factors did not differ between the males or females in the validation and derivation cohorts in this study. This shows that blood correction provides improved precision in native myocardial T1, but not to the extent that it completely eliminates sex differences in clinical patients, even though the remaining difference had a very small magnitude. Further studies may be of value to better understand these mechanisms.

### MOLLI sequences and myocardial T1 variability

Several different methods to quantitatively measure T1 relaxation times are currently in clinical use [37]. The original MOLLI pulse sequence with acquisition over 17 successive heart beats has become a commonly used T1 mapping method [1]. The original MOLLI sequence has high precision, however the accuracy declines with longer T1 values, and is particularly susceptible to higher heart rates [38]. Variations of MOLLI have been purposed to reduce breath-hold duration, and reduce heart rate sensitivity such as the 5(3s)3 [30, 39] scheme. The 5(3s)3 protocol has high precision, but somewhat limited accuracy for shorter T1 values [38]. The Shortened MOLLI (ShMOLLI) scheme consists of sequential inversion-recovery acquisitions of nine successive heart beats [29] and uses a conditional fitting algorithm to account for the short recovery period between inversion pulses. The conditional fitting discards data, sacrificing precision without improving accuracy [38]. T1 mapping can also be performed with saturation recovery single-shot acquisition (SASHA) [40] which mitigates the underestimation of T1 values by MOLLI, however with a reduced precision [38]. SASHA was modified to mitigate heart rate dependency with saturation pulse prepared heart-rate-independent inversion recovery (SAPPHIRE) [41], however, this approach still has a lower precision in measuring myocardial T1 compared to MOLLI [42]. The myocardial T1 values in the patients in our study were 1023 ± 42.6 ms and 1030 ± 45.2 ms, which closely corresponds to the myocardial T1 found in the healthy volunteers in our study (1027 ± 37 ms), both acquired using a MOLLI sequence. However, this is longer and more variable than 962 ± 25 ms in healthy volunteers imaged with ShMOLLI [10]. The mean differences in T1 are related to the inherent differences between the sequences, and the differences in variability are likely related to differences in ROI placement strategy. Furthermore, the blood correction model did not decrease myocardial T1 variability in healthy volunteers, however it did eliminate gender differences. This is probably due to fact that healthy volunteers have a greater homogeneity in myocardial T1 values, but also blood T1 values. It is possible that the resultant blood correction reduction, even in a study powered to reduce variability in healthy volunteers, would be so small that it’s not clinically relevant. Therefore, the necessary study design to illustrate the clinical performance of blood correction in native T1 mapping is to study patients with variable T1 in both blood and myocardium.

Furthermore, T1 values differ over CMR magnetic field strength, both for blood and myocardium. Blood correction assuming linear correlation has been performed successfully at 3 T, however the changes in variability following blood correction were not evaluated [36]. Therefore, blood correction is feasible for 3 T as well, however the optimal correction model would have to be evaluated in dedicated clinical cohorts and healthy volunteers.

### Clinical implications

The proposed validated blood correction method might have clinical significance for diseases that are characterized by a change in native myocardial T1 values, such as amyloidosis, iron-overload cardiomyopathy, Anderson-Fabry disease and other non-ischemic heart diseases. With increased diagnostic precision, these diseases could potentially be identified earlier when only subtle differences in the myocardial T1 may be present. The range of myocardial T1 values was decreased by 25% in the derivation cohort. This suggests that blood correction helps in determining the true biological variability in myocardial T1 values, making it easier to differentiate pathological myocardial T1 values from normal myocardial T1 values. Native T1 mapping has shown diagnostic promise in amyloidosis [5, 43], as native myocardial T1 increases with amyloid deposition and inflammation [44]. Amyloidosis can be apparent in late gadolinium enhancement (LGE) images, but the patterns are often diffuse and globally distributed [45]. This can pose a challenge when selecting the inversion time for LGE imaging, since normal myocardium may be absent. However, native T1 mapping is not dependent upon normal myocardium for determining normality, and can therefore provide a useful imaging method in amyloidosis. Furthermore, native myocardial T1 mapping has been shown to be an independent predictor of survival in amyloidosis [46]. By correcting for myocardial blood content, the differences between normal and diseased myocardium might increase and allow for earlier clinical detection of disease.

Anderson-Fabry is a disease with accumulation of glycosphingolipids within lysosomes, causing progressive fibrosis in certain tissues [47]. Native T1 mapping has shown promising results for identifying these patients since native myocardial T1 decreases, and might be used as a surrogate measure for the disease [7]. With less variability in native myocardial T1 values following blood correction, diagnostic precision increases. Therefore, the diagnostic accuracy provided by native T1 mapping of cardiac involvement by Anderson-Fabry disease may improve.

Furthermore, differences in myocardial T1 values at RV insertion points between patients with and without pulmonary hypertension (PH) were highlighted by blood correction [36], suggesting that blood correction can increase diagnostic precision for diseases with more subtle changes in native myocardial T1.

## Limitations

This study included patients with known or suspected heart disease to develop and validate a blood correction method. Patients were used in order to yield the clinical ranges of myocardial and blood T1 values necessary for determining meaningful correlations. As patients were used, this also sometimes resulted in changes in the MOLLI acquisition parameters with varying FoV size and phase encoding lines, which can influence the accuracy of the T1 measurement. However, the strategy used underscores that the resulting blood correction model is valid for clinical use, since adjusting image acquisition parameters is a part of the clinical routine. Furthermore, only septal myocardial T1 values were acquired, and not all segments according to American Heart Association (AHA) segmentation scheme, which has been used in other studies [36, 48]. Notably, it is not known how the blood correction model in this study affects the rest of the myocardium. However, Reiter et al. [22] studied all segments of the left ventricle and identified negligible differences in myocardial T1 throughout the left ventricle. This suggests that the same blood correction could be applied for the entire left ventricular myocardium.

## Conclusions

Blood correction improves the precision of native myocardial T1 measurement by 13% and reduces gender differences. Image-based correction of native myocardial T1 values for R1 and R1* of blood is valid for clinical use and may improve disease detection in the clinical evaluation of native myocardial T1, and reduce sample size needs for clinical research.

## Abbreviations

AHA: American Heart Association
BSA: Body surface area
bSSFP: Balance steady state free precession
CMR: Cardiovascular magnetic resonance
ECV: Extracellular volume fraction
EDV: End diastolic volume
EF: Ejection fraction
ESV: End systolic volume
FoV: Field of view
LGE: Late gadolinium enhancement
LV: Left ventricle
LVM: Left ventricular mass
MBV: Myocardial blood volume
MOLLI: Modified Look-Locker inversion recovery
PAT: Parallel acquisition technique
ROI: Region of interest
RV: Right ventricle
SD: Standard deviation
SV: Stroke volume
TE: Echo time
TR: Repetition time
